# CXCL14 Maintains hESC Self-Renewal through Binding to IGF-1R and Activation of the IGF-1R Pathway

**DOI:** 10.3390/cells9071706

**Published:** 2020-07-16

**Authors:** Chih-Lun Cheng, Shang-Chih Yang, Chien-Ying Lai, Cheng-Kai Wang, Ching-Fang Chang, Chun-Yu Lin, Wei-Ju Chen, Po-Yu Lin, Han-Chung Wu, Nianhan Ma, Frank Leigh Lu, Jean Lu

**Affiliations:** 1 Graduate Institute of Life Sciences, National Defense Medical Center, Taipei 114, Taiwan; Skyclear1120@gmail.com (C.-L.C.); hcw0928@gate.sinica.edu.tw (H.-C.W.); 2 Genomics Research Center, Academia Sinica, Taipei 115, Taiwan; david503@gate.sinica.edu.tw (S.-C.Y.); believe614@hotmail.com (C.-Y.L.); kiki75277527@yahoo.com.tw (C.-K.W.); g3930901@gate.sinica.edu.tw (C.-F.C.); michelle11770@gmail.com (C.-Y.L.); d02b48011@ntu.edu.tw (W.-J.C.); Adam80829@gmail.com (P.-Y.L.); 3 Institute of Biochemistry and Molecular Biology, National Yang-Ming University, Taipei 112, Taiwan; 4 Genome and Systems Biology Degree Program, College of Life Science, National Taiwan University, Taipei 106, Taiwan; 5 Institute of Cellular and Organismic Biology, Academia Sinica, Taipei 115, Taiwan; 6 Department of Biomedical Sciences and Engineering, National Central University, Taoyuan City 320, Taiwan; nianhan.ma@g.ncu.edu.tw; 7 Department of Pediatrics, National Taiwan University Children’s Hospital, National Taiwan University Hospital, and National Taiwan University Medical College, Taipei 100, Taiwan; 8 National Core Facility Program for Biotechnology, National RNAi Platform, Taipei 112, Taiwan; 9 Department of Life Science, Tzu Chi University, Hualien 970, Taiwan; 10 Graduate Institute of Medical Sciences, National Defense Medical Center, Taipei 114, Taiwan

**Keywords:** CXCL14, BRAK, BMAC, Mip-2γ, human embryonic stem cell, self-renewal, cell cycle, IGF-1R

## Abstract

Human embryonic stem cells (hESCs) have important roles in regenerative medicine, but only a few studies have investigated the cytokines secreted by hESCs. We screened and identified chemokine (C-X-C motif) ligand 14 (CXCL14), which plays crucial roles in hESC renewal. CXCL14, a C-X-C motif chemokine, is also named as breast and kidney-expressed chemokine (BRAK), B cell and monocyte-activated chemokine (BMAC), and macrophage inflammatory protein-2γ (MIP-2γ). Knockdown of *CXCL14* disrupted the hESC self-renewal, changed cell cycle distribution, and further increased the expression levels of mesoderm and endoderm differentiated markers. Interestingly, we demonstrated that CXCL14 is the ligand for the insulin-like growth factor 1 receptor (IGF-1R), and it can activate IGF-1R signal transduction to support hESC renewal. Currently published literature indicates that all receptors in the CXCL family are G protein-coupled receptors (GPCRs). This report is the first to demonstrate that a CXCL protein can bind to and activate a receptor tyrosine kinase (RTK), and also the first to show that IGF-1R has another ligand in addition to IGFs. These findings broaden our understanding of stem cell biology and signal transduction.

## 1. Introduction

Human embryonic stem cells (hESCs), isolated from the inner cell mass of blastocysts, are widely known for their unique abilities for unlimited self-renewal and pluripotency [1,2]. These two important potentials enable hESCs to generate vast quantities of multiple different tissue cells (except placental cells), which sheds substantial light on regenerative medicine and stem cell biology [3,4].

The characteristics of hESCs are regulated by many essential factors, such as intracellular transcriptional regulation and extracellular cytokine stimulation [5,6,7]. Cytokines can establish an extensive microenvironment to support the properties of hESCs [8,9,10]. Most cytokines, such as basic fibroblast growth factor (bFGF), epidermal growth factor (EGF), and insulin-like growth factor (IGF), are secreted from feeder layers or supplemented in commercial media that support hESC renewal [8,11]. In contrast, only a few studies have noted that hESCs also can secrete autocrine cytokines such as bFGF, growth differentiation factor-3 (GDF-3), transforming growth factor β (TGFβ) and activin, which are essential for hESC self-renewal and survival [8,10,12,13]. However, studies of hESC-secreted cytokines and their mechanisms require further exploration.

Chemokines are a type of cytokine first identified in immune cells, and they participate in the immune response, cell migration, and cell proliferation [14,15,16,17]. Chemokines can be divided into four classes (C, C-C, C-X-C, and C-X3-C) based on the number of cysteine residues on their N-terminal chain, and the spacing of the first two cysteine residues with respect to one or more intervening amino acids [18]. All currently identified chemokine receptors are G protein-coupled receptors (GPCRs), which induce the activation of cAMP and trigger downstream signal transduction in the mitogen-activated protein kinase (MAPK) and phosphoinositide 3-kinase (PI3K)-AKT pathways to mediate cell mobilization [19]. Previously, the C–X–C motif ligands (CXCL) CXCL1 (GROα), and CXCL8 (IL-8), secreted from human feeder cells, have been reported to be beneficial for the self-renewal and pluripotency of hESCs [20,21]. CXCL1, CXCL2, and CXCL8 can sustain hESC growth through CXCL receptor 2 (CXCR2) in the absence of bFGF supplementation [21]. The CXCR2-mTOR axis mediates the renewal status of hESCs in association with *β-catenin*, while knockdown of *CXCR2* leads to mesendoderm differentiation of hESCs [22]. These investigations indicated that CXCL proteins play indispensable roles in the regulation of hESC characteristics. However, whether hESCs secrete CXCL proteins to maintain their renewal status is unknown.

*CXCL14*, a 13 kDa chemokine, is involved in the progression and metastasis of many malignant cells, such as prostate cancer, breast cancer, and lung carcinoma cells [23,24,25]. CXCL14 has been suggested to compete for the binding of CXCL12 to its receptor, CXCR4, and block signaling activation stimulated by CXCL12 [26]. However, in another paper, the authors used *CXCR4*-transfected HEK293 cells to claim that CXCL14 could not inhibit the activation of CXCL12-induced CXCR4 phosphorylation [27]. These authors concluded that the CXCL14 pathway may depend on an unknown CXCL14 receptor [27]. To date, no prior study has reported the functions of CXCL14 in hESCs, and the corresponding receptor for CXCL14 stimulation in hESCs is still unknown.

In this study, we found that CXCL14, a chemokine secreted by hESCs, can mediate the self-renewal of hESCs. This was investigated by screening the phosphorylation profile of 49 human receptor tyrosine kinases (RTKs) with an RTK array after stimulating the cells with CXCL14. Furthermore, we provided the first demonstration that CXCL14 can bind the insulin-like growth factor 1 receptor (IGF-1R), and thus activate the IGF-1R signaling pathway to support hESC renewal. Our study is the first to prove that IGF-1R has other novel ligands in addition to IGFs and that the CXCL protein can be a ligand for RTKs.

Overall, these findings significantly contribute to the knowledge about chemokine modulation in stem cell biology and reveal a novel role of chemokines in RTK signal transduction.

## 2. Materials and Methods

The methods followed the standard guidelines. All culture reagents, unless specified, were obtained from Thermo Fisher Scientific (Waltham, MA, USA). All chemicals, unless specified, were purchased from Sigma-Aldrich (St. Louis, MO, USA).

### 2.1. Cell Lines and Culture Conditions

The hESC H9 line was provided by WiCells (Madison, WI, USA) (Thomson et al., 1998). The other hESC line, HUES6 (S6), was obtained from Harvard University (Dr. Douglas A. Melton, Boston, MA, USA) (Cowan et al., 2004). Both hESC lines were maintained in Dulbecco’s modified Eagle’s medium (DMEM)/F12 supplemented with 20% knockout serum replacement (KSR), 1% nonessential amino acids, 2 mM L-glutamine, 0.1 mM 2-mercaptoethanol (Sigma-Aldrich), and 8 ng/mL human bFGF and were cultured on C57BL/6 MEFs. For feeder-free culture conditions, culture plates were coated with Matrigel matrix (BD Biosciences, San Jose, CA, USA) to replace feeder cells, and hESCs were cultured with feeder-free conditioned medium for all the experiments. For detecting the phosphorylation in 15 min, cells were cultured in DMEM/F12 only with CXCL14 and IGF1. HEK293T cells were maintained in DMEM supplemented with 10% inactivated fetal bovine serum (FBS) (Biological Industries, Cromwell, CT, USA). All cells were cultured in a 37 °C incubator with 5% CO_2_. For embryonic body (EB) formation assay, hESCs were digested with enzyme and cultured in DMEM with 10% FBS on a low attachment plate. The EB will be generated spontaneously within 5 days.

### 2.2. Plasmids

Control shRNA targeting red fluorescent protein (*shRFP*, TRCN000007220) was acquired from the National RNAi Core Facility (Taipei, Taiwan). shRNAs targeting *CXCL14* (*shCXCL14-1*, TRCN0000057923; and *shCXCL14-2*, TRCN0000057924) and *IGF-1R* (*shIGF-1R-1*, TRCN0000121192; and *shIGF-1R-2*, TRCN0000121193) were also obtained from the National RNAi Core Facility. For the CRISPR/Cas9 genome editing method, sgRNA sequences (Table S1) were designed by an online CRISPR design program (crispr.mit.edu) and clone into a pgRNA-CKB vector (Addgene, Plasmid #73501).

### 2.3. Lentivirus Transfection and hESC Infection

To produce lentivirus for hESC infection, HEK293T cells were seeded on a 6-well plate (1 × 10^6^ cells /well). On next day, HEK293T were transfected with 4 μg of lentiviral vectors containing 2 μg of targeted shRNA, 1.8 μg of pCMVR8.91, and 0.2 μg of pMDG (National RNAi Core Facility, Taipei, Taiwan) with Turbofect transfection reagent (Thermo Fisher Scientific). After 24 h, the medium was substituted with DMEM containing 1% BSA and 10% FBS. After 48 h and 72 h, the supernatants were harvested. For infection, hESCs were seeded in a 6-well plate (1 × 10^5^ cells per well) with the feeder-free system and subsequently treated with virus [multiplicity of infection (MOI) = 10] and protamine sulfate (8 μg/mL) (P3369; Sigma-Aldrich) for 16 h. On the next day, cells were selected by 2 μg/mL puromycin (P8833; Sigma-Aldrich).

### 2.4. RNA Purification and Quantitative Real-Time PCR (qRT-PCR)

RNA was extracted by the standard RNeasy Micro (Qiagen, Hilden, Germany) protocol. RNA (1 μg/sample) was treated with DNase and reverse transcribed with SuperScript III (Thermo Fisher Scientific). For qRT-PCR, cDNA (20 ng/set) was combined with SYBR Green 2× master mix (KAPA Biosystems, Wilmington, MA, USA). The relative cDNA amounts were measured and quantified using an ABI 7900 Real-Time PCR System (Applied Biosystems, Carlsbad, CA, USA). The relative expression levels of mRNA were analyzed by normalization to glyceraldehyde 3-phosphate dehydrogenase (GAPDH) expression levels by calculating the comparative Ct values. All the primer sequences were listed in Table S1.

### 2.5. Western Blotting and Phospho-RTK Array

Total protein was extracted with lysis buffer (20 mM Tris, 1% Triton X-100, and 150 mM NaCl) supplemented with 1 mM Na_3_VO_4_ and protease inhibitors. After quantification, 30 μg of protein lysates were segregated by SDS-PAGE and transferred on the PVDF membranes. Then, membranes were blocked with 3% BSA-TBST for 1 h. Next, membranes were incubated with antibodies for 16 h at 4 °C. After membranes were washed with TBST (150 mM NaCl, 0.1 M Tris, and 0.1% of Tween 20), they were treated with secondary antibodies for 1 h at room temperature. Membranes were exposed to Western Chemiluminescent HRP Substrate (WBKLS0500; Millipore, Darmstadt, Germany), and signals were detected with a UVP Imaging System (ChemiDoc-It, Sopachem Life Science, Nazareth, Belgium). All antibodies are listed in Table S2. The Human Phospho-RTK Array Kit (Proteome Profiler Antibody Array, R&D Systems, Minneapolis, MN, USA) was used following the manufacturer’s guidelines.

### 2.6. Immunofluorescence (IF) Staining

Cells were fixed with 4% formaldehyde for 10 min. After washing with PBS, cells were permeabilized with Triton X-100 (0.3%) for 10 min and washed again with PBS. Then, cells were incubated with antibodies in PBS containing 3% BSA for 16 h at 4 °C. After washing with PBS, cells were incubated with Alexa Fluor-conjugated secondary antibodies (Thermo Fisher Scientific) for 1 h at 37 °C (light-protected). 4′,6-Diamidino-2′-phenylindole dihydrochloride (DAPI) (0.5 μg/mL) (D9542; Sigma-Aldrich) was used for nuclei staining. All images were acquired with the immunofluorescence microscope (Zeiss Axiovert 200M, Carl Zeiss Light Microscope, Göttingen, Germany) and visualized by SPOT software 5.1 (SPOT IMAGING^TM^, Sterling Heights, MI, USA). All antibodies were listed in Table S2.

### 2.7. Enzyme-Linked Immunosorbent Assay (ELISA)

To measure the concentrations of secreted CXCL14, we followed the manufacturer’s instructions (Broster Human CXCL14 ELISA kit, Tools, Taipei, Taiwan). Supernatants were collected on day 6 after shRNA expression in hESCs. A total of 100 μL of each sample and CXCL14 standard solution were added to a precoated 96-well plate. The sample was incubated at 37 °C for 90 min. After the contents were removed, 100 μL of anti-CXCL14 antibody working solution was added and incubated at 37 °C for 60 min. After the plate was washed with PBS, 100 μL of prepared ABC working solution was added to each sample and incubated at 37 °C for 30 min. Next, the sample was washed with PBS 5 times, and 90 μL of TMB color-developing agent was added to the samples and incubated at 37 °C for 20–25 min (in the dark). When the blue color was visible in some wells, 100 μL of prepared TMB stop solution was added to the wells, and the absorbance was then detected at 450 nm by a microplate reader (Bio-Rad Benchmark Plus^TM^, Hercules, California, USA) in 30 min. All procedures followed the standard protocol.

For the in vitro protein interaction assay, 50 ng of BSA, IGF-1Rα-His (Sino Biological, Wayne, PA, USA), CXCL14 (PeproTech, New Jersey, USA) or IGF-1 (PeproTech), in coating buffer (0.42 g NaHCO_3_ in 50 mL double-distilled (dd) water) was added to the wells and incubated at 4 °C overnight. The next day, each well was washed with 100 μL of PBST 3 times and blocked with 1% BSA in PBS for 1 h. After CXCL14 or IGF-1Rα-His was added for interaction analysis for 1 h at RT temperature, the wells were again washed with PBST 3 times and incubated with specific antibodies (1:1000 dilution in blocking buffer) for 1 h at room temperature. Then, the samples were washed with PBST 3 times, and secondary antibodies were added (diluted 1:1000 in blocking buffer) for 1 h at room temperature. After 3 washes with PBST, 90 μL of TMB solution was added to the wells and incubated at 37 °C in the dark until shades of blue were visible in the wells. Then, 100 μL of TMB stop solution was added to the wells, and the absorbance at 450 nm was determined by a microplate reader (Bio-Rad benchmark Plus^TM^, CA, USA) within 30 min. For the competition assay, an ELISA plate was coated with IGF-1Rα-His at 4 °C overnight, incubated with 0.4 μM CXCL14 protein (5 μg/mL) and cotreated with or without different concentrations (0, 0.4, 2, and 4 μM) of IGF-1 protein for 1 h at room temperature. After 3 washes with PBST and incubation with the anti-CXCL14 antibody for 1 h at room temperature, secondary antibody and the TMP development assay were performed as above.

### 2.8. Flow Cytometry

For PI staining, cells were fixed with 70% ethanol. After 3 washes with PBS, cells were stained with PI substrate (20 μg/mL) and RNase (200 μg/mL) for 1 h. The cells were analyzed using a FACSCanto^TM^ instrument (Becton Dickinson, Franklin Lakes, NJ, USA), and the results were processed and analyzed by FASCDiva^TM^ (BD Biosciences) software.

### 2.9. Co-Immunoprecipitation (Co-IP)

Cells were lysed in lysis buffer (the same buffer used for Western blot analysis). Cell lysates (1 mg) were incubated with 1 μg antibodies or isotype IgG control overnight at 4 °C. Mag-Beads Protein G (5 μL) (Tools, Taipei, Taiwan) was added and further incubated at 4 °C for 4 h to capture antibody-bound proteins. All samples were washed with PBST (1.47 mM KH_2_PO_4_, 7.7 mM Na_2_HPO_4_, 137 mM NaCl, 2.7 mM KCl, and 0.1% of Tween 20) 5 times, denatured with 2× SDS sample buffer (5% 2-mercaptoethanol) and analyzed by Western blotting. All antibodies are listed in Table S2.

### 2.10. Duolink Proximity Ligation Assay (PLA)

A Duolink In Situ kit (Sigma-Aldrich) was used to detect protein interactions by fluorescence imaging. Cells were fixed as the protocol of immunofluorescence staining. According to the standard protocol, samples were incubated with blocking solution at 37 °C for 1 h and subsequently incubated with diluted primary antibody at 4 °C overnight. Wells were washed with washing buffer A 2 times for 5 min each. A diluted PLA probe was added to each sample and incubated at 37 °C for 1 h. Next, samples were washed twice with washing buffer A. Then, the diluted ligation reagent was added and incubated at 37 °C for 30 min. After 2 washes for 5 min each with washing buffer A, polymerase solutions were incubated with the samples at 37 °C for 100 min. Then, the wells were washed with washing buffer B 2 times for 10 min each and further washed with 0.01% washing buffer B for 1 min. Finally, the samples were mounted with mounting reagents containing DAPI. After a 15 min incubation, images were acquired with an immunofluorescence microscope (Zeiss Axiovert 200 M, Carl Zeiss Light Microscope, Göttingen, Germany) and visualized by SPOT software 5.1 (SPOT IMAGING^TM^, Sterling Heights, MI, USA). The antibodies used are listed in Table S2.

### 2.11. Knockout CXCL14 in Inducible CRISPR iPSC Line

The inducible CRISPR iPSC line (CRISPRn) was kindly provided by Bruce R. Conklin’s lab [28]. We did not adopt any human tissue directly from human. The experiments were approved by Human Subjects Research Ethics (Academia Sinica, Taipei, Taiwan). The ethical code is AS-IRB01-18023 (2018/05/02-2023/12/31). To generate *CXCL14* knockout cells, CRISPRn cells were first seeded in a 12-well plate (1.5 × 10^5^ per well). On the next day, the medium was changed with fresh StemFlex medium (A3349401; Thermofisher) without doxycycline (as a solvent control) or with doxycycline (2 μM) to induce the expression of Cas9 for further 24 h. The cells were transfected with a pair of sgRNAs by TransIT^®^-LT1 Transfection Reagent (MIR2300; Mirus) on day 3. After 24 hrs, cells were cultured in E8 medium with Blasticidin (2.5 μg/mL) on the first day and with Blasticidin (5 μg/mL) on the third day to the fifth day with or without doxycycline to select cells. The selected cells were seeded in a 96-well plate (1 cell per well) and cultured with E8 medium to get the clones of *CXCL14* knockout cells.

### 2.12. Statistical Analysis

All the repeat experiments were performed at least in 3 different samples for statistical analysis. All the statistical results are presented as the means ± standard deviations (SD). Student’s t-test and ANOVA were used for the statistical analysis, and significance was set at * *P* < 0.05, ** *P* < 0.01, and *** *P* < 0.001.

## 3. Results

### 3.1. CXCL14 Expression is Enriched in Undifferentiated hESCs

**Figure 1:**
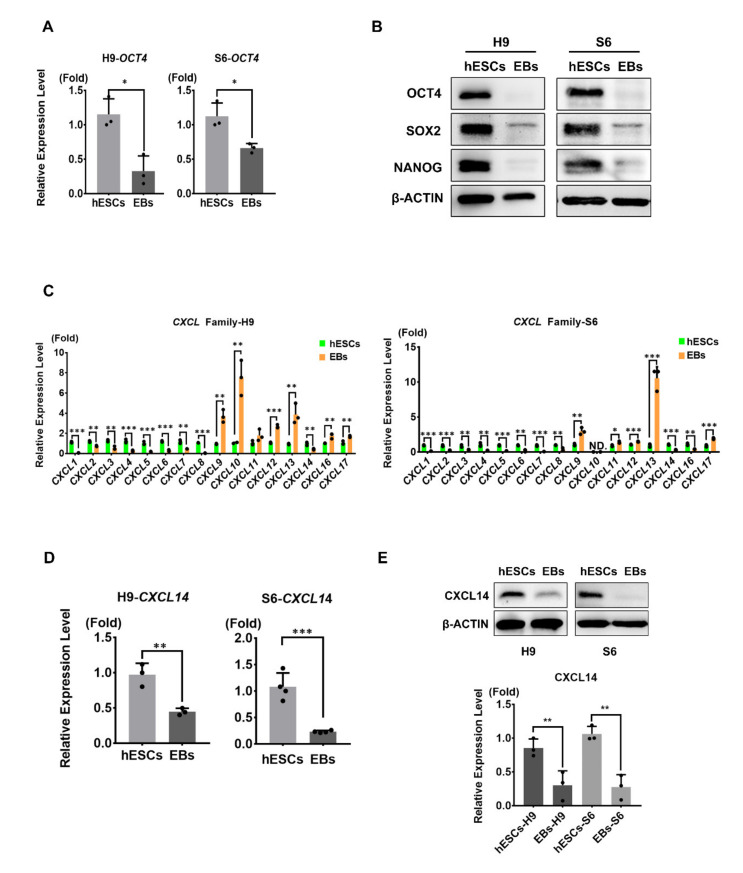
*CXCL14* (*C-X-C motif Ligand 14*) expression is enriched in undifferentiated human embryonic stem cells (hESCs). Embryonic bodies (EBs) are hESCs differentiated for 5 days. (**A**) qRT-PCR (Quantitative Real-Time PCR) analysis of the self-renewal marker *OCT4* (*Octamer-binding transcription factor 4*) expression in both H9 and S6 undifferentiated hESCs and differentiated EBs. The data were normalized to *GAPDH* (*Glyceraldehyde-3-phosphate dehydrogenase*), and the error bars represent the standard deviations of three replicates. * *P* < 0.05, Student’s t-test. (**B**) Western blot analysis and the quantification of OCT4, SOX2 (SRY-Box transcription factor 2) and NANOG (Homeobox protein NANOG) protein expression levels in undifferentiated hESCs and EBs. β-ACTIN was applied as the internal control. The error bars represent the standard deviations of three replicates. ** *P* < 0.01, *** *P* < 0.001, Student’s t-test. ND, the data was nondetectable. (**C**) All C-X-C motif chemokines were analyzed by qRT-PCR in undifferentiated H9/S6 hESCs and EBs. All qRT-PCR data were normalized to *GAPDH*. The error bars represent the standard deviations of three replicates. ** *P* < 0.01, *** *P* < 0.001, Student’s t-test. (**D**) The mRNA expression levels of *CXCL14* were analyzed by qRT-PCR in both H9 and S6 undifferentiated hESCs and EBs. The data were normalized to *GAPDH*. The error bars represent the standard deviations of three and four replicates. ** *P* < 0.01, *** *P* < 0.001; Student’s t-test. (**E**) Western blot analysis and the quantification of CXCL14 protein expression levels in undifferentiated hESCs and EBs. β-ACTIN was applied as the internal control. The error bars represent the standard deviations of three replicates. ** *P* < 0.01, Student’s t-test.

The CXCL family of chemokines has many biological functions and plays crucial roles in stem cell growth [21,29]. To investigate the roles of chemokines in pluripotency, we evaluated the expression levels of chemokines in undifferentiated and differentiated hESCs. We spontaneously differentiated two different hESC lines [H9 and HUES6 (S6)] by embryonic body (EB) formation. By comparing the undifferentiated and differentiated hESCs, we first checked the differentiation state by analyzing the mRNA expression levels of the crucial self-renewal marker *OCT4* which were specifically downregulated in EBs (Figure 1A). In addition, we further confirmed the differentiation status by observing the protein expression level of self-renewal markers, OCT4, SOX2, and NANOG (Figure 1B). Then, we analyzed the mRNA expression levels of all C-X-C motif chemokines between the undifferentiated hESCs and differentiated EBs of H9 and S6 hESCs. The expression of *CXCL1-8* and *CXCL14* was downregulated in EBs compared to that in undifferentiated hESCs (H9 and S6) (Figure 1C). In contrast, *CXCL9-13*, *CXCL16*, and *CXCL17* were upregulated in differentiated hESCs (H9) (Figure 1C). However, the expression level of CXCL10 was non-detectable and CXCL16 was downregulation in differentiated hESCs (S6) (Figure 1C). In previous studies, CXCL1-8 were reported to be secreted by feeder cells, and CXCL1 and CXCL8 were involved in the self-renewal of hESCs [21]. Via quantitative real-time PCR (qRT-PCR) and Western blot analyses, we further confirmed that a chemokine, CXCL14, is enriched in both undifferentiated hESCs (H9 and S6) and depleted in differentiated EBs (Figure 1D,E). These results indicated that CXCL14 expression is enriched in undifferentiated hESCs.

### 3.2. CXCL14 is Secreted by hESCs and is Essential for hESC Self-Renewal

**Figure 2:**
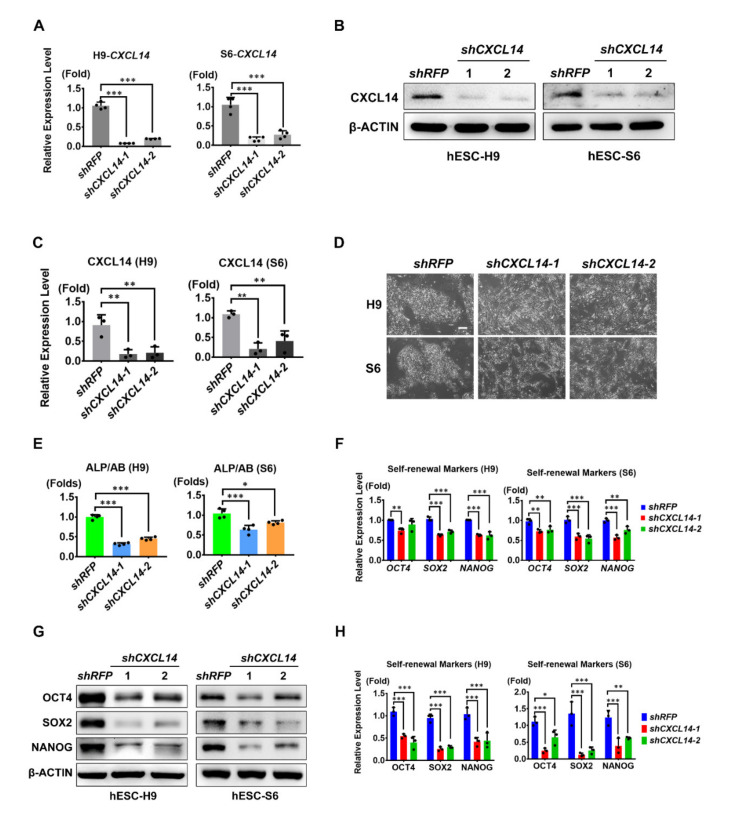
Knockdown of *CXCL14* disrupts hESC self-renewal. Both H9 and S6 hESCs were infected with *shRFP* or *shCXCL14* for 6 days. (**A**) qRT-PCR analysis of the *CXCL14* knockdown efficiency in two independent *shCXCL14* clones of H9 and S6 cells. All error bars represent the standard deviations of four replicates. The significance level was set at *** *P* < 0.001; ANOVA (Dunnett’s multiple comparison test). (**B**) Western blot analysis of CXCL14 expression levels in *shRFP*- and *shCXCL14*-expressing hESC H9 and S6 cells. (**C**) Quantification of Western blot analysis B. The error bars represent the standard deviations of three replicates. The significance level was set at ** *P* < 0.01; ANOVA (Dunnett’s multiple comparison test). (**D**) Gross morphology of shRNA-expressing H9 and S6 cells. The scale bar represents 100 μm. (**E**) An alkaline phosphatase (ALP) assay was performed to examine stem cell marker expression. The ALP values were normalized to the relative cell number (Alamar blue assay, AB). The error bars represent the standard deviations of four replicates. The significance level was set at * *P* < 0.05, *** *P* < 0.001; ANOVA (Dunnett’s multiple comparison test). (**F**) qRT-PCR analysis of *OCT4*, *SOX2*, and *NANOG* mRNA expression levels in both H9 and S6 hESCs expressing *shRFP* or *shCXCL14*. The error bars represent the standard deviations of three replicates. The significance levels were set at ** *P* < 0.01 and *** *P* < 0.001; ANOVA (Dunnett’s multiple comparison test). (**G**) Western blot analysis of OCT4, SOX2, and NANOG protein expression levels in shRNA-infected hESCs. β-ACTIN was applied as the internal control. (**H**) Quantification of Western blot analysis G. The error bars represent the standard deviations of three replicates of H9 and S6 cells. The significance level was set at * *P* < 0.05, ** *P* < 0.01, and *** *P* < 0.001; ANOVA (Dunnett’s multiple comparison test).

To further investigate whether CXCL14 is involved in the regulation of hESC self-renewal, we downregulated the expression of *CXCL14* by two independent *shCXCL14* clones. Six days after infection with *shCXCL14*, the mRNA expression of *CXCL14* was significantly downregulated in both H9 and S6 hESCs (Figure 2A). The Western blot analysis results showed that the protein expression of CXCL14 was also downregulated in both hESC lines (Figure 2B,C). Because CXCL14 is a secreted protein, we also performed an enzyme-linked immunosorbent assay (ELISA) to measure the protein content in the culture medium. While CXCL14 was easily detected in the supernatant of the *shRFP* control group, it could not be detected in the culture supernatant of *CXCL14* knockdown H9 hESCs (Figure S1). Moreover, in both H9 and S6 hESCs, *CXCL14* knockdown induced dramatic morphological changes (Figure 2D). Although dome-shaped colonies were formed by *shRFP*-infected hESCs, *shCXCL14* cells exhibited a fibroblastic phenotype (Figure 2D). These data suggested that *shCXCL14*-expressing cells were differentiated.

We also found that in H9 and S6 hESCs, *CXCL14* knockdown decreased alkaline phosphatase and Alamar (ALP/AB) ratio, an ESC self-renewal marker, in *shCXCL14*-infected H9 and S6 hESCs (Figure 2E). Also, the crucial self-renewal markers *OCT4*, *SOX2*, and *NANOG* were downregulated at both the mRNA and protein levels in *shCXCL14*-infected hESCs (Figure 2F–H). Furthermore, by measuring the expression levels of differentiated markers, we showed that *CXCL14* depletion indeed led to hESC differentiation. The mRNA expression results showed significant upregulation of *TWIST*, *SNAIL2*, *GATA4*, *GATA6*, *SOX7*, and *SOX17*, which are mesoderm and endoderm lineage markers (Figure S2A). However, the expression of ectoderm markers such as *SOX1* and *PAX6* was downregulated in hESCs after *CXCL14* knockdown (Figure S2A). Taken together, these results indicated that knockdown of *CXCL14* disrupts the self-renewal of hESCs and leads to the differentiation.

### 3.3. Exogenous CXCL14 Maintains the Disruption of Self-Renewal Mediated by shCXCL14

**Figure 3:**
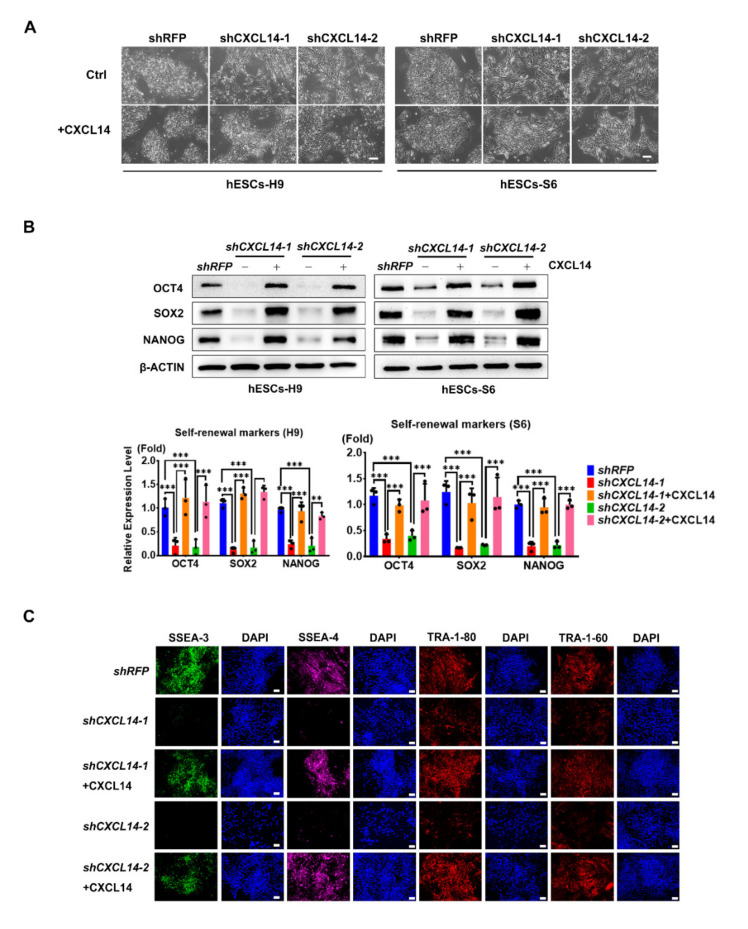
Exogenous CXCL14 maintains the disruption of self-renewal mediated by *shCXCL14*. Cells were treated with recombinant CXCL14 protein (100 ng/mL) for 5 days. (**A**) Morphology of shCXCL14-expressing H9 and S6 cells cotreated with recombinant CXCL14 protein. (**B**) Western blot analysis and quantification of OCT4, SOX2, and NANOG protein expression levels in *shRFP*- or *shCXCL14*-infected H9 and S6 cells with or without CXCL14 treatment. β-ACTIN was applied as the internal control. The error bars represent the standard deviations of three replicates of H9 and S6 cells. The significance level was set at ** *P* < 0.01 and *** *P* < 0.001; ANOVA (Tukey’s multiple comparison test). (**C**) Immunofluorescence assay of the self-renewal markers stage-specific embryonic antigen (SSEA)-3 (green), SSEA-4 (purple), TRA-1-80 (red), and TRA-1-60 (red) in *shRFP*- or *shCXCL14*-expressing hESCs with or without CXCL14 treatment. Cell nuclei were stained with DAPI (blue). The scale bar represents 100 μm.

To exclude off-target effects of shRNAs, we used recombinant CXCL14 protein to restore the disrupted self-renewal of *shCXCL14*-infected hESCs. In *shCXCL14*-infected H9 and S6 hESCs that had knockdown endogenous *CXCL14*, the addition of exogenous CXCL14 proteins in the culture supernatants maintained the cell morphology and protein expression levels of the self-renewal markers OCT4, SOX2, and NANOG upon CXCL14 activity in both *shCXCL14*-infected H9 and S6 cells (Figure 3A,B and Figure S2B). The immunofluorescence (IF) staining also indicated the downregulation of hESC surface markers stage-specific embryonic antigen (SSEA)-3, SSEA-4, TRA-1-80 and TRA-1-60 in shCXCL14-infected cells were all maintained by CXCL14 treatment (Figure 3C). Consistently, the protein expression levels of the differentiation markers GATA binding protein (GATA) 4, SRY-box transcription factor (SOX) 7, and SOX17 were decreased after the maintaining of self-renewal by CXCL14 treatment (Figure S2C). Taken together, these results showed the maintaining effects of CXCL14 treatment on *shCXCL14*-infected hESCs.

### 3.4. CXCL14 Regulates Cell Cycle Progression in hESCs

**Figure 4:**
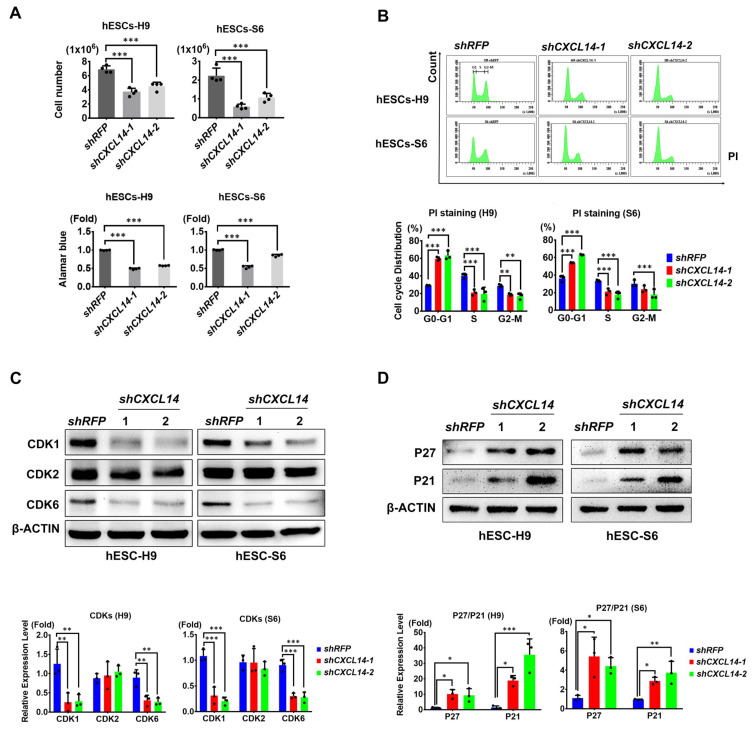
Knockdown of *CXCL14* affects cell cycle progression in hESCs. (**A**) Trypan blue exclusion assay and Alamar blue (AB) assay for cell number counting and cell viability after shCXCL14 infection in both H9 and S6 hESCs. The error bars represent the standard deviations of four replicates. The significance level was set at *** *P* < 0.001; ANOVA (Dunnett’s multiple comparison test). (**B**) Propidium iodide (PI) staining with flow cytometry in shRNA-expressing H9 and S6 cells. Quantification data showing the distribution of shRNA-infected hESCs in G0-G1 phase, S phase and G2-M phase. The error bars represent the standard deviations of three replicates. The significance levels were set at ** *P* < 0.01 and *** *P* < 0.001; ANOVA (Dunnett’s multiple comparison test). (**C**) Western blot analysis and quantification of the cell cycle regulators CDK1 (Cyclin-dependent kinase 1), CDK2, and CDK6 in shRNA-expressing H9 and S6 cells. β-ACTIN was applied as the internal control. The error bars represent the standard deviations of three replicates. The significance levels were set at ** *P* < 0.01 and *** *P* < 0.001; ANOVA (Dunnett’s multiple comparison test). (**D**) Western blot analysis and quantification of the cell cycle inhibitors P27, and P21 in shRNA-expressing H9 and S6 cells. β-ACTIN was applied as the internal control. The error bars represent the standard deviations of three replicates. The significance levels were set at * *P* < 0.05, ** *P* < 0.01, and *** *P* < 0.001; ANOVA (Dunnett’s multiple comparison test).

Regarding the regulation of CXCL14 on cell growth, the cell counting results showed significant decrease in the cell number and cell viability of *shCXCL14*-infected H9 and S6 cells (Figure 4A). Hence, we considered that CXCL14 may affect cell growth in hESCs. First, Propidium iodide (PI) staining was performed to investigate cell cycle progression in hESCs. In both *shCXCL14*-infected hESC lines, the number of cells in G0-G1 phase was significantly increased, while the number of cells in S phase was decreased (Figure 4B). No sub-G1 phase was increased in *shCXCL14*-expressing cells (Figure 4B). Then, we analyzed the proliferation markers, Ki-67 and phosho-HISTONE H3 (pH3) with IF staining, the proliferative ability of hESCs were significantly decreased in *shCXCL14*-infected cells (Figure S3A). Furthermore, the addition of recombinant CXCL14 protein restored the cell cycle distribution in both the *shCXCL14*-expressing hESC lines; the numbers of cells in the G0-G1 and S phases were returned to normal (Figure S3B,C). The Ki-67 and pH3 analysis also showed the maintenance effects of exogenous CXCL14 treatment on cell proliferative ability of *shCXCL14*-infected hESCs (Figure S3A). Moreover, we investigated cell cycle-associated markers, the cyclin-dependent kinases (CDKs), by Western blot analysis. The protein expression of cyclin-dependent kinase (CDK) 1 and CDK6 was downregulated in both H9 and S6 hESCs with *CXCL14* knockdown (Figure 4C). However, no significant difference was observed in the expression levels of CDK2 in hESCs with *CXCL14* knockdown (Figure 4C). Consistent with these findings, the expression of the cell cycle inhibitors P27 and P21 was upregulated after *CXCL14* depletion in both hESC lines (Figure 4D). To investigate the sequence of pluripotency and cell cycle, we also examined the self-renewal markers in relation to cell cycle post *shRFP* and *shCXCL14* infection. The downregulation of self-renewal markers was evident by Western blot analysis as early as day 2 (Figure S4A,B). In contrast, the cell cycle distribution was no difference between *shRFP*- and *shCXCL14*-expressing cells (Figure S4C,D). Hence, the changing of cell cycle might be the result of cell differentiation in *shCXCL14*-infected hESCs. Taken together, knockdown of *CXCL14* impacted proliferation and cell cycle distribution in hESCs.

### 3.5. CXCL14 Binds and Activates IGF-1R in hESCs

**Figure 5:**
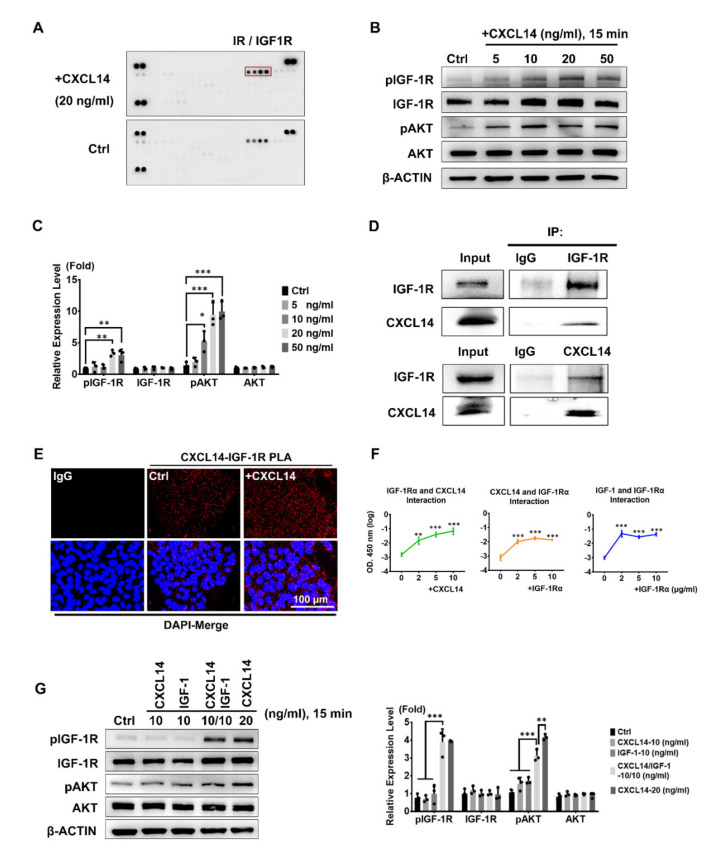
CXCL14 binds to the IGF-1R (Insulin-like growth factor 1 receptor) in hESCs. (**A**) RTK (Receptor Tyrosine Kinase) array analysis to determine phosphorylation patterns in hESCs treated with CXCL14 (20 ng/mL) for 15 min. (**B**) Western blot analysis for the phosphorylation pattern of IGF-1R and its downstream effector AKT after cells were treated with CXCL14 (0, 5, 10, 20, or 50 ng/mL) in DMEM/F12 only for 15 min. β-ACTIN was applied as the internal control. (**C**) Quantification of Western blot analysis B. The error bars represent the standard deviations of three replicates. The significance levels were set at * *P* < 0.05, ** *P* < 0.01, and *** *P* < 0.001; ANOVA (Dunnett’s multiple comparison test). (**D**) Co-immunoprecipitation analysis of the CXCL14 and IGF-1R interaction. Isotype IgG was used as negative control. (**E**) Duolink PLA (Proximity Ligation Assay) assay for analyzing the interaction of CXCL14 and IGF-1R in vivo. Red fluorescence indicates the interaction of these two proteins; cell nuclei were stained with DAPI (blue). IgG was the isotype negative control. The interaction of endogenous CXCL14 with IGF-1R was investigated as the control (Ctrl). Cells treated with exogenous CXCL14 (+CXCL14) were also monitored. The scale bar represents 100 μm. (**F**) ELISA for detecting the direct interaction of purified CXCL14, IGF-1, and IGF-1Rα in vitro by precoating the plate with IGF-1Rα (2 μg/mL). Different concentrations of CXCL14 (μg/mL) were added (green, left). ELISA assay by precoating the plate with CXCL14 (2 μg/mL). Different concentrations of IGF-1Rα (μg/mL) were added (yellow, middle). ELISA for detecting the direct interaction of purified IGF-1 and IGF-1Rα in vitro by precoating the plate with IGF-1 (2 μg/mL). Different concentrations of IGF-1Rα (μg/mL) were added (blue, right). The error bars represent the standard deviations of four and five replicates. The significance level was set at ** *P* < 0.01 and *** *P* < 0.001; ANOVA (Dunnett’s multiple comparison test). (**G**) Western blot analysis and quantification of the phosphorylation pattern of IGF-1R and AKT after cells were treated with CXCL14 and/or IGF-1 for 15 min cells. Β-ACTIN was applied as the internal control. The error bars represent the standard deviations of three replicates. The significance levels were set at ** *P* < 0.01 and *** *P* < 0.001; ANOVA (Dunnett’s multiple comparison test).

From the current knowledge, the role of the CXCL14 receptor in hESCs is still unclear. We sought to reveal the mechanism of CXCL14 in hESC self-renewal and hypothesized that CXCL14 could bind to a cell surface receptor and then trigger the corresponding signaling cascade to support hESC renewal. Therefore, we used a human phospho-RTK array to identify the possible receptor by CXCL14 stimulation. After 15 min of stimulation, CXCL14 activated the phosphorylation of IGF-1R/IR, as evidenced by the spots in the RTK array (Figure 5A). Western blot analysis further confirmed that exogenous CXCL14 treatment dose-dependently increased the phosphorylation of IGF-1R and its downstream effector AKT (Figure 5B,C).

Since the phosphorylation of IGF-1R was induced by CXCL14 at 15 min, we investigated whether IGF-1R could be the receptor for CXCL14. Therefore, we conducted Co-immunoprecipitation (Co-IP) to analyze the interaction between CXCL14 and IGF-1R. When we used primary antibodies to pull down IGF-1R or CXCL14 from hESC protein lysates, we could detect interaction signals for the CXCL14 and IGF-1R proteins by Western blot analysis (Figure 5D). This result indicates the interaction between CXCL14 and IGF-1R. To further confirm the interaction in vivo, we demonstrated the interaction by Duolink proximity ligation assay (PLA) with IF staining. The interaction signals for CXCL14 and IGF-1R could be detected and were further amplified by the PLA probes. The amplified RFP signals could be observed in hESCs compared with those in the Control-IgG group, demonstrating that endogenous CXCL14 and IGF-1R interacted with each other (Figure 5E, Ctrl). Moreover, when we treated hESCs with exogenous CXCL14, the increase in the RFP signal suggested enhanced CXCL14 binding to IGF-1R (Figure 5E, +CXCL14).

To elucidate whether purified CXCL14 can directly interact with IGF-1Rα (the extracellular domain), in vitro ELISA was performed. We precoated an ELISA plate with purified IGF-1Rα and then incubated the plate with purified CXCL14 and anti-CXCL14 antibody and found that the optical density (OD) values were increased in a dose-dependent manner (Figure 5F, left). This result suggests the possibility of direct interaction between IGF-1R and CXCL14. Consistent with this finding, when the plate was coated with CXCL14, the OD value also increased after the application of IGF-1Rα (Figure 5F, middle). Similarly, incubation with IGF-1 and IGF-1Rα enhanced the ELISA readout in a dose-dependent manner (Figure 5F, right). To further study the effects of CXCL14 and IGF-1 on the IGF-1R pathway, we treated hESCs with CXCL14 and IGF-1 separately or in combination to examine the downstream signals. The co-treatment synergistically activates the downstream signals while compare to cells treating with CXCL14 or IGF-1 alone (Figure 5G). The result indicated a synergistic effect of CXCL14 and IGF-1 on IGF-1R signaling pathway in hESCs (Figure 5G). Collectively, the results indicated that CXCL14 can bind to IGF-1R and activate downstream signal transduction in hESCs.

### 3.6. CXCL14 Regulates hESC Self-Renewal Through the Activation of the IGF-1R Signaling Pathway

**Figure 6:**
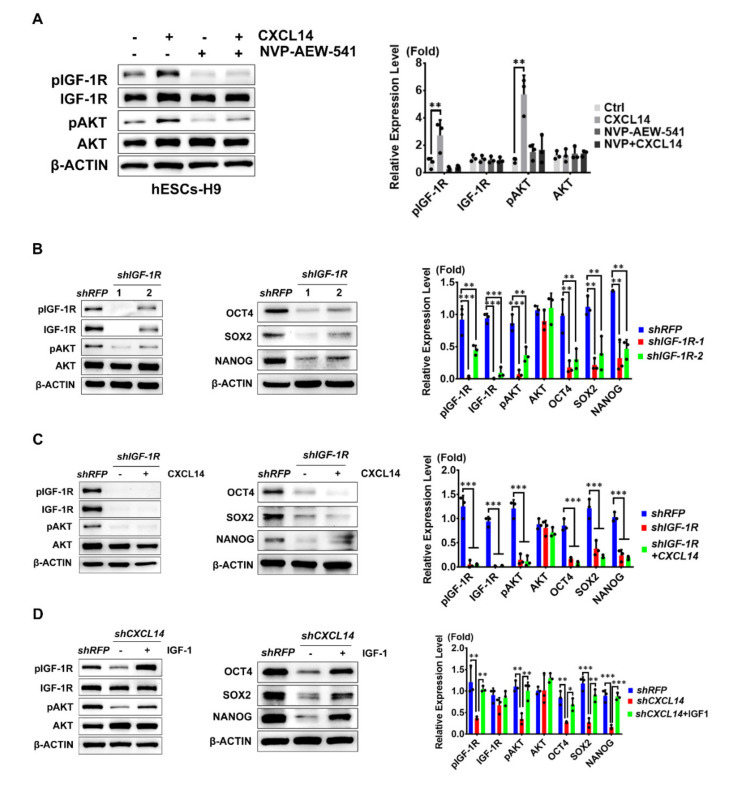
CXCL14 regulates hESC self-renewal through the activation of the IGF-1R signaling pathway. (**A**) Western blot analysis and quantification for detecting IGF-1R and AKT phosphorylation in H9 cells treated with CXCL14 (20 ng/mL) with or without the IGF-1R inhibitor (NVP-AEW-541, 2 μM) for 15 min. (**B**) Western blot analysis and quantification of IGF-1R-AKT phosphorylation and self-renewal markers OCT4, SOX2, and NANOG in *shIGF-1R*-infected hESCs. (**C**) Western blot analysis and quantification of IGF-1R-AKT phosphorylation and self-renewal markers OCT4, SOX2, and NANOG in *shIGF-1R*-infected hESCs cotreated with or without CXCL14 (100 ng/mL). (**D**) Western blot analysis and quantification of IGF-1R-AKT phosphorylation and self-renewal markers OCT4, SOX2, and NANOG in *shCXCL14*-infected hESCs cotreated with or without IGF-1 (100 ng/mL). β-ACTIN was applied as the internal control for all Western blot analyses. All the error bars represent the standard deviations of three replicates. The significance level was set at * *P* < 0.05, ** *P* < 0.01, *** *P* < 0.001; ANOVA (Dunnett’s multiple comparison test).

To further examine whether CXCL14 could activate signals without activating IGF-1R signaling cascades for hESC self-renewal, we treated hESCs with an IGF-1R inhibitor (NVP-AEW 541). The phosphorylation of IGF-1R and AKT was stimulated by CXCL14 treatment, while NVP-AEW 541 treatment abolished the activation of the IGF-1R signaling pathway mediated by CXCL14 (Figure 6A). Next, to further confirm that CXCL14 supports hESC self-renewal through the IGF-1R signaling pathway, we disrupted IGF-1R expression by two independent shRNA clones. The successful knockdown of *shIGF-1R* was demonstrated by Western blot analysis and the phosphorylation of IGF-1R and downstream AKT was downregulated in *shIGF-1R*-expressing hESCs (Figure 6B). Interestingly, *shIGF-1R*-expressing hESCs also decreased the protein expression levels of OCT4, SOX2, and NANOG (Figure 6B). Then, we examined whether the regulatory ability of CXCL14 is via the IGF-1R signaling pathway. When we knocked down the receptor, *IGF-1R*, the effect of CXCL14 activity on AKT activation was abolished (Figure 6C). Moreover, CXCL14 was no longer maintaining the expression levels of the self-renewal markers *OCT4*, *SOX2*, and *NANOG* while the IGF-1R was knocked down (Figure 6C).

According to these results, we further hypothesized that IGF-1, an IGF-1R ligand, can activate IGF-1R signaling and support the self-renewal of *CXCL14* knockdown hESCs. In support of this hypothesis, the decrease in IGF-1R and AKT phosphorylation in *shCXCL14*-infected hESCs was restored by the IGF-1 stimulation (Figure 6D). We also demonstrated that in *shCXCL14*-infected hESCs, treatment with IGF-1 restored the expression levels of three critical self-renewal markers, OCT4, SOX2, and NANOG (Figure 6D). 

Taken together, these results indicated that CXCL14 maintains hESC self-renewal through the activation of the IGF-1R signaling pathway.

### 3.7. Knockout of CXCL14 Impairs Self-Renewal of hPSCs

To further exclude the off-target of shRNA, we knocked out *CXCL14* in hPSCs by inducible CRISPR/Cas9 genome editing method [28]. Both Western blot and immunofluorescence staining assay indicated the complete depletion of CXCL14 protein expression in two *CXCL14^-/-^* cell lines (Figure S5A,B). Similar to the knockdown effects of *shCXCL14*-expressing hESCs, knockout *CXCL14* also disrupted the activation of IGF-1R-AKT signaling pathway and the expression levels of self-renewal markers OCT4, SOX2, and NANOG (Figure S5C,D). Taken together, our results suggested that knockout of *CXCL14* disrupted the self-renewal ability in hPSCs.

## 4. Discussion

**Figure 7:**
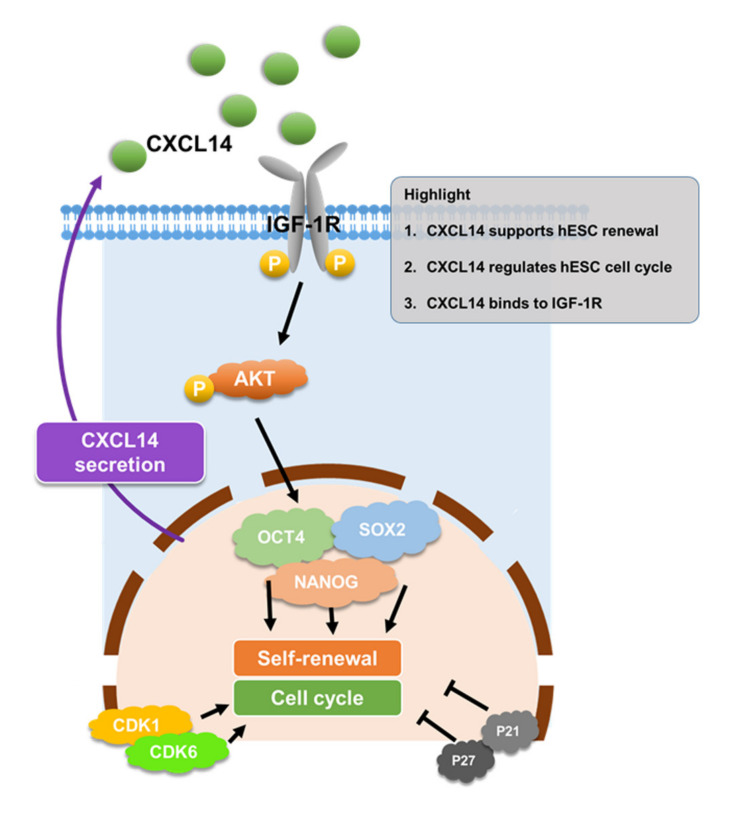
CXCL14 regulates hESC self-renewal and cell cycle through IGF-1R signaling pathway. A summary shows the cell signaling events in this study. In short, hESCs can release CXCL14, which further binds to the IGF-1R of hESCs. CXCL14 activates the signaling activation of IGF-1R-AKT axis to support the expressions of self-renewal markers, OCT4, SOX2, and NANOG. CXCL14 also regulates the cell cycle distribution and the expression of cell cycle markers, CDK1, CDK6, P21, and P27 in hESCs.

The cytokines and chemokines in the hESC environment maintain the self-renewal ability of stem cells [29]. Only a few cytokines, such as bFGF, TGF-β, and GDF3, have been demonstrated as autocrine factors of hESCs [8,10,13]. In this study, a C-X-C motif chemokine, CXCL14, was demonstrated to maintain the self-renewal and pluripotency abilities of hESCs. CXCL14 expression was enriched in undifferentiated hESCs, and the expression level was decreased upon hESC differentiation (Figure 1). Knockdown of *CXCL14* disrupted the expression levels of the self-renewal markers OCT4, SOX2, and NANOG, which further resulted in hESC differentiation (Figure 2 and Figure S2). In addition, CXCL14 depletion decreased the cell number, reduced cell proliferation, and changed the cell cycle distribution of hESCs (Figure 4 and Figure S3). To exclude off-target effects of shRNA, recombinant CXCL14 protein added to the culture medium was provided to restore the self-renewal status, recover the proliferative ability, and alter the cell cycle distribution of *shCXCL14*-infected hESCs (Figure 3, Figures S2 and S3). Interestingly, CXCL14 bound directly to IGF-1R and activated its downstream effector AKT (Figure 5 and Figure 6). Consistent with these findings, knockdown of *IGF-1R* inhibited hESC self-renewal, whereas IGF-1 can restore the knockdown phenotype of *shCXCL14* (Figure 6). To further rule out the possibility of off-target effect by using shRNA, we established the *CXCL14* knockout hPSCs according to the inducible CRISPR/Cas9 genome editing method [28]. Consistent with the phenomenon of *shCXCL14*-infected hESCs, the results of *CXCL14^-/-^* cells also shown the downregulation of self-renewal markers and inactivation of IGF-1R-AKT signaling pathway (Figure S5). In summary, we elucidated that CXCL14 can maintain hESC self-renewal through the activation of the IGF-1R signaling pathway (Figure 7).

Chemokines are well known for their chemoattractant functions in many cell types [16,30,31]. In recent years, niche-dependent chemokines have been shown to maintain the renewal status of stem cells [20,21,29]. Most cytokines and chemokines that regulate hESC renewal are secreted by feeder cells [mouse embryonic fibroblasts (MEFs) or human fibroblasts] or supplemented in commercial media [20,32]. Human placental cells, as feeder cells, can release CXCL1, CXCL2, and CXCL8 to mediate the self-renewal and pluripotency of hESCs through CXCR2 [21]. In addition, both hESCs and human induced pluripotent stem cells (hiPSCs) can secrete CXCL1, CXCL2, CXCL8, and CXCL10 and support cell migration and self-renewal [29]. However, whether additional chemokines are involved in the regulation of hESC growth was unclear. Our results showed that hESCs can express all CXCL proteins and that the expression of CXCL1-CXCL8 and CXCL14 was enriched in both undifferentiated hESCs (Figure 1). In contrast, the mRNA expression of *CXCL9*, *CXCL10*, *CXCL11*, *CXCL12*, and *CXCL13* was upregulated in differentiated hESCs (EBs) (Figure 1). Similar to our results, one report showed by chemokine array screening that CXCL8, CXCL10, and CXCL12 were secreted in the culture supernatant of hESCs [29]. However, this article did not assess whether these chemokines were autocrine factors and also there is no investigation about the potential mechanisms by further analysis. Thus, chemokine regulation in hESC self-renewal remains a great mystery. Here, we found that the chemokine CXCL14 is secreted by hESCs and uncovered the roles of CXCL14 in self-renewal maintenance. The discovery of the detailed mechanisms by which additional chemokines participate in hESC renewal will need further study.

In recent years, CXCL14 has been shown to modulate the regulation of cellular trafficking, immune responses, cell metabolism, and tumor progression in different cell types [17,30,33]. CXCL14 promotes the progression and metastasis of breast cancer, lung cancer, and pancreatic cancer [23,25,34]. However, the receptor for CXCL14 remains to be elucidated. In human leukemia-derived cell lines and CD34^+^ hematopoietic progenitor cells, CXCL14 can compete with CXCL12 and thus inhibit CXCL12-CXCR4 signaling [26]. In 2014, Otte et al. demonstrated in *CXCR4*-transfected HEK293 and Jurkat T cells that CXCL14 could not bind CXCR4 and inhibit CXCL12-CXCR4 signal transduction [27]. Recent studies have suggested that the G protein-coupled receptor GPR85 and atypical chemokine receptor 2 (ACKR2) could be the target of CXCL14 in breast cancer cells [34,35]. Indeed, the binding of CXCL14 to GPR85 has been demonstrated [35]. Activation of GPR85 by CXCL14 triggered ERK1/2 and AKT pathway activation, which promoted breast cancer cell progression [35]. CXCL14 also stimulated ACKR2 to mediate tumor invasion and metastasis. However, no direct interaction of CXCL14 and ACKR2 was found in further investigation by these authors [34]. According to those studies, the receptors and the signal transduction pathways mediated by CXCL14, which are complicated and cell-type dependent processes, may be different from those of conventional C-X-C-type chemokines [36]. Our investigation identified a novel CXCL14 receptor model in hESCs. By analyzing protein interactions both in vivo and in vitro (via Duolink PLA staining, Co-IP, and ELISA), we confirmed that CXCL14 bound to IGF-1R, an RTK, and can subsequently activate downstream signal transduction (Figure 5 and Figure 6). This report is the first to indicate that IGF-1R is a CXCL14 receptor, and to identify a CXCL receptor that is not a G protein-coupled receptor. Previously, only IGFs were recognized as ligands for IGF-1R. This evidence may contribute to new knowledge leading to the discovery of other chemokine and receptor pairings in hESCs and other cell types.

IGF-1R, a heterotetrameric RTK consisting of two α and β form subunits, is important for cell growth and differentiation in many cell types [37,38]. IGF-1R was indicated to be strongly associated with hESC growth through the activation of IGF-1 from medium supplementation or secretion by the feeder layers [32,39]. A reduction in IGF-1R-PI3K-AKT pathway activity resulted in the differentiation and poor proliferation of hESCs [32]. The binding of IGFs to IGF-1R was found to support hESC growth and survival [32,40]. The expression level of IGF-1R was reduced by miR-223 during hESC differentiation [41]. Signaling downstream of IGF-1R involves the phosphorylation of PI3K-AKT, which was reported to contribute to the self-renewal of undifferentiated hESCs [42].

However, no prior study has indicated whether IGF-1R knockdown in hESCs affects the expression levels of self-renewal markers. Here, we found that CXCL14 bound to IGF-1R and activated downstream AKT signaling to support hESC self-renewal (Figure 5 and Figure 6). Furthermore, knockdown of *IGF-1R* in hESCs disrupted self-renewal status (Figure 6). Similar to previous investigations, this study demonstrated that the IGF-1R signaling pathway is important for hESC self-renewal and pluripotency. Moreover, an IGF-1R inhibitor blocked the activation of the IGF-1R-AKT signaling pathway triggered by CXCL14 treatment, and the addition of CXCL14 treatment did not restore the self-renewal status of *shIGF-1R*-expressing hESCs (Figure 6). This evidence indicated that CXCL14 mediates hESC self-renewal through binding to IGF-1R and the activation of the IGF-1R signaling pathway. Notably, IGF-1-stimulated IGF-1R signaling activation rescued the phenotypes associated with *CXCL14* knockdown in hESCs (Figure 6). The co-treatment of CXCL14 and IGF-1 could enhance the activation of IGF-1R signals (Figure 5). Therefore, this finding may suggest that these two proteins may have different binding sites on IGF-1R to support hESC self-renewal.

Chemokines and chemokine receptors have been studied to regulate growth and cell cycle progression in different cell types [43,44,45]. In hESCs, a shortened G1 phase can maintain self-renewal, and a high proliferation rate is required for hiPSC reprogramming [46,47]. Therefore, we investigated cell growth and cell cycle distribution in hESCs by adopting a gain- and loss-of-function approach. Our results revealed that *CXCL14* knockdown reduced the expression levels of CDK1 and CDK6 but increased the expression levels of the cell cycle inhibitors, P27 and P21 (Figure 4). The increase in G0-G1 arrest and the truncation of S phase in *shCXCL14*-infected hESCs indicated the reduction in rapid hESC self-renewal (Figure 4). According to previous investigations, G0-G1 arrest of hESCs was induced by the downregulation of CDKs and upregulation of P27 and P21, which were highly related to the loss of hESC renewal [48,49]. CDK6 was reported to regulate the G1-S transition, and CDK1 downregulation was reported to impair hESC self-renewal and promote hESC differentiation [48,50], similar to our results in hESCs with knockdown of *CXCL14* (Figure 4). Disruption of CDK2 also causes G1 arrest, loss of pluripotency, and upregulation of P21 and P27 [51]. However, a further investigation from the same group also suggested that CDK2 plays a role in the modulation of hESC apoptosis [52]. On the other hand, hESCs with knockdown of *CHAC2*, an essential hESC gene, also exhibited a decrease in CDK2 expression, accompanied by apoptosis [53]. According to these studies, the expression levels of CDK2 were not significantly decreased in *shCXCL14*-expressing hESCs, eliciting the downregulation of CXCL14 and possibly facilitating the differentiation of hESCs rather than inducing apoptosis through CDK2 downregulation. Consistent with this reason, PI staining demonstrated no sub-G1 phase was increased in *shCXCL14*-expressing cells (Figure 4). The IF staining also indicated the downregulation of proliferation markers Ki-67 and pH3 in *shCXCL14*-infected cells (Figure S3). Based on this evidence, knockdown of *CXCL14* impacted cell proliferation but may not affect apoptosis in hESCs.

To establish a suitable microenvironment for hESC growth and self-renewal maintenance, the balance of chemokines, cytokines, and other growth factors is indispensable [11,29,54]. In this study, we not only investigated the critical roles of CXCL14 in hESC coordination, but also found a novel link between CXCL14 and IGF-1R in signal transduction. The CXCL14-IGF-1R axis may also apply to the physiological regulation of and mechanistic studies in other cell types. Thus, our research contributes to the current knowledge of hPSCs and the signal transduction of chemokines.

## Supplementary Materials

The following are available online at https://www.mdpi.com/2073-4409/9/7/1706/s1, Figure S1: ELISA assay for in vivo secretion of CXCL14 and in vitro assay for interaction, Figure S2: Knockdown of *CXCL14* promotes hESC differentiation, Figure S3: Exogenous CXCL14 restores the cell cycle distribution of *CXCL14* knockdown hESCs, Figure S4: Downregulation of CXCL14 regulates hESC cell cycle through hESC differentiation, Figure S5: Knockout of *CXCL14* impairs self-renewal of hPSCs, Table S1: qRT-PCR and sgRNA primer list, Table S2: Primary antibody list.
