# Supplementary material for: CXCL14 Maintains hESC Self-Renewal through Binding to IGF-1R and Activation of the IGF-1R Pathway
Source: Cells. 2020 Jul 16;9(7):1706. doi: 10.3390/cells9071706 (PMC7407311; doi:10.3390/cells9071706)
Supplement: Supplementary file 1 [file cells-09-01706-s001.pdf]

Figure S1

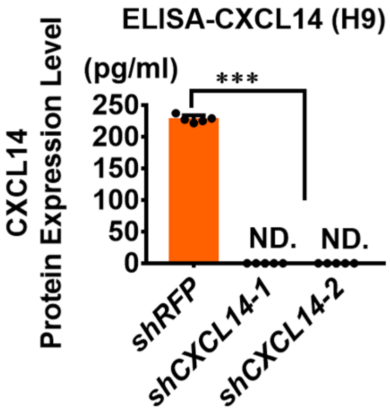

Figure S1. Enzyme-Linked Immunosorbent (ELISA) assay for in vivo secretion of CXCL14. ELISA assay for the level of secreted CXCL14 in the culture supernatant of shRFP-, shCXCL14-1-, and shCXCL14-2-infected hESC-H9 cells. The error bars represent the standard deviations of five replicates. The significance level was set at *** P <0.001; ANOVA (Dunnett's multiple comparison test). ND, the data were non-detectable.

Figure S2

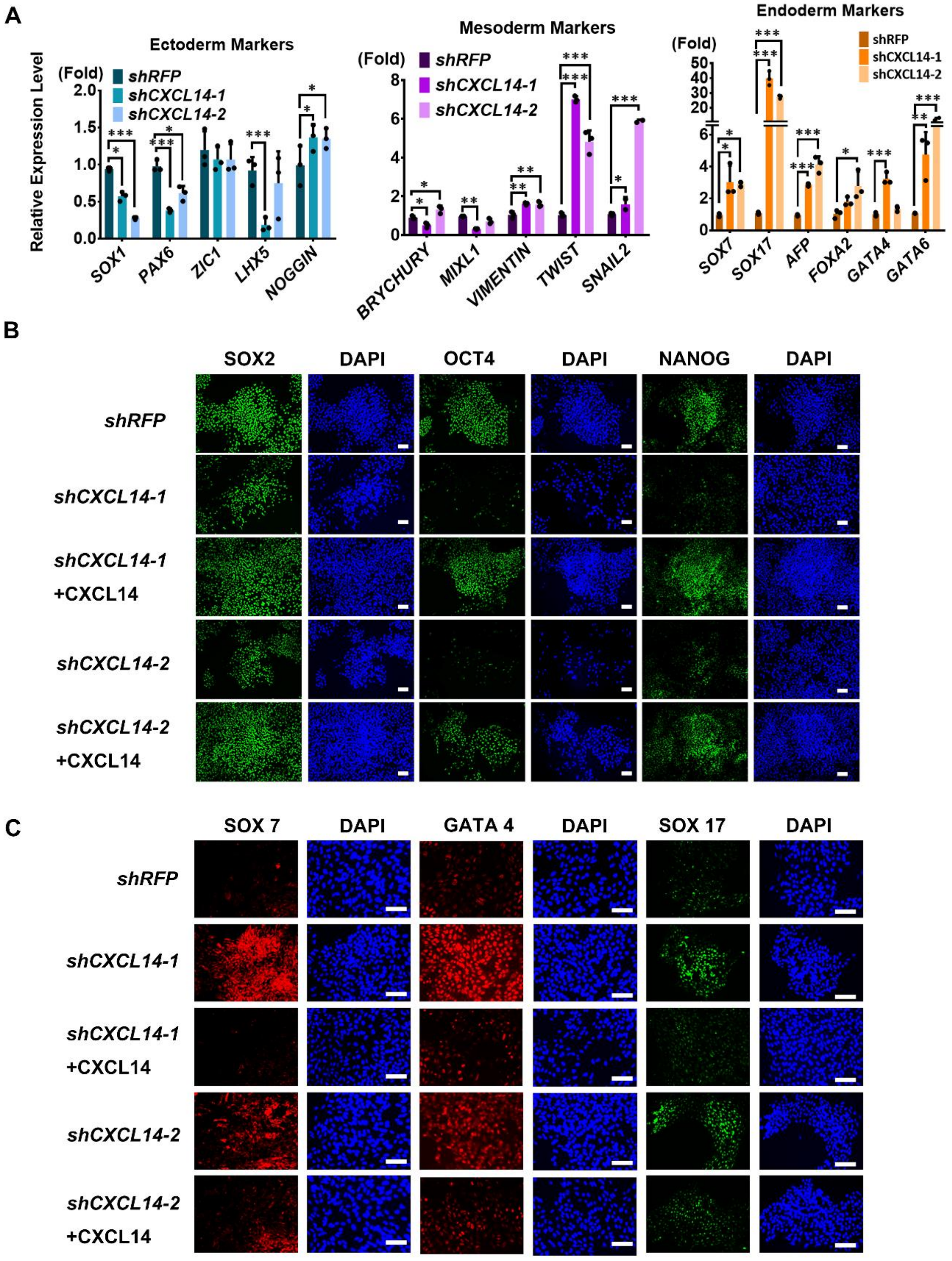

Figure S2. Knockdown of *CXCL14* promotes hESC differentiation. (**A**) qRT-PCR analysis of the differentiation markers for the three germ layers ectoderm, mesoderm, and endoderm in shRNA-expressing hESCs. The expression data were normalized to *GAPDH*, and all error bars represent the standard deviations of three replicates. The significance level was set at * $P < 0.05$, ** $P < 0.01$, *** $P <$ 0.001; ANOVA (Dunnett's multiple comparison test). (**B**) Immunofluorescence staining for the self-renewal markers SOX2 (green), OCT4 (green), NANOG (green). Cell nuclei were stained with DAPI

(blue). The scale bar represents 100 μm. Cells were treated with recombinant protein CXCL14 (100 ng/ml) for rescue experiments. **(C)** Immunofluorescence staining for the differentiated markers SOX7 (red), GATA4 (red), and SOX17 (green) in shRNA-expressing hESCs. Cell nuclei were stained with DAPI (blue). The scale bar represents 100 μm. Cells were treated with recombinant protein CXCL14 (100 ng/ml) for rescue experiments. SOX7 (SRY-box transcription factor 7), GATA4 (GATA binding protein 4), SOX17 (SRY-box transcription factor 17)

Figure S3

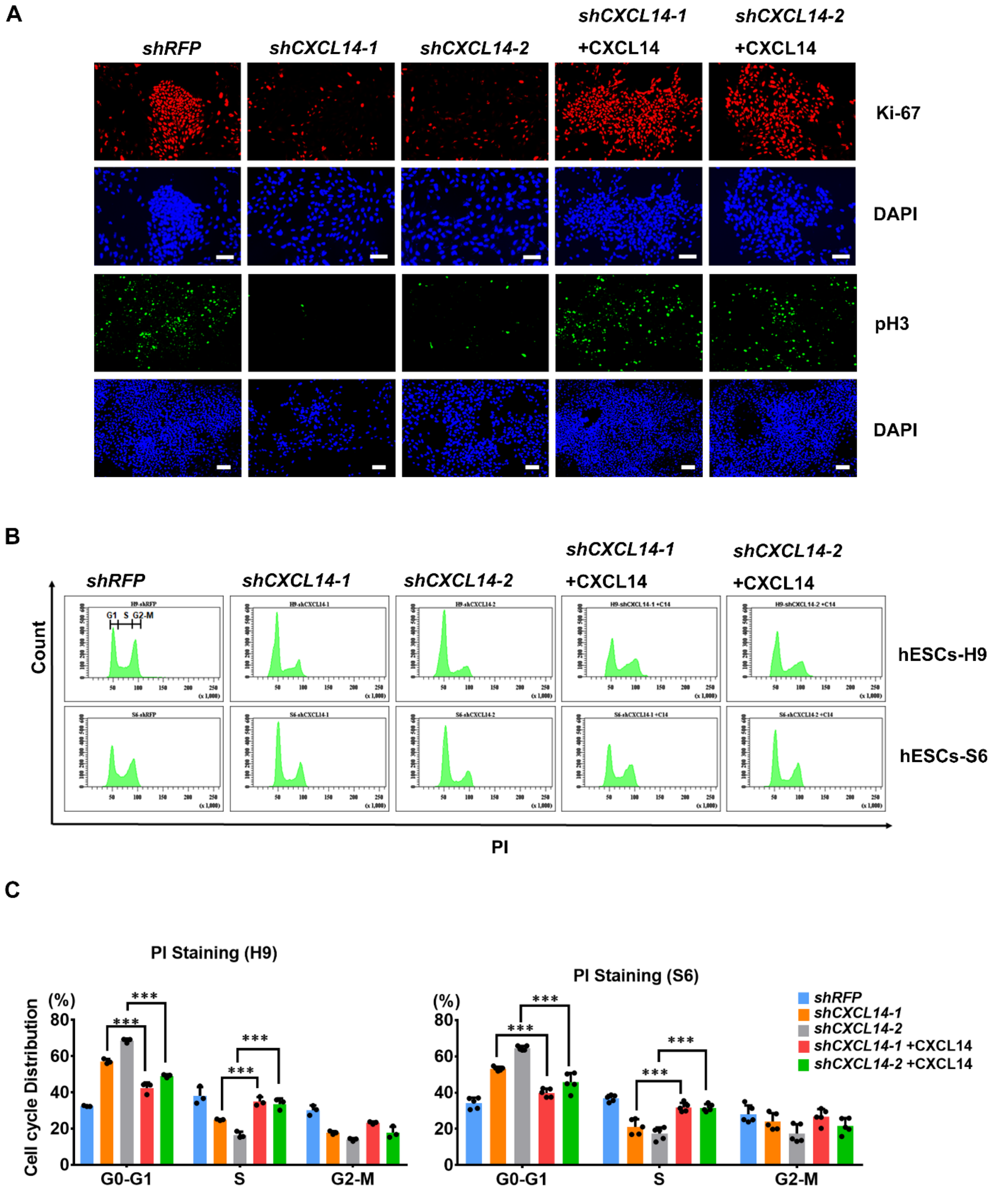

Figure S3. Exogenous CXCL14 restores the cell cycle distribution of *CXCL14* knockdown hESCs. (**A**) Immunofluorescence staining for the Ki-67 (red) protein and phospho-HISTONE H3 (green) in shRNA-expressing hESCs treated with or without CXCL14 (100 ng/ml). Cell nuclei were stained with DAPI (blue). The scale bar represents 100 μm. **(B**) Cell cycle analysis of shRNA-infected H9 and S6 cells treated with or without CXCL14 (100 ng/ml). **(C**) Quantification data showing the distribution of G0-G1-phase, S-phase and G2-M-phase of *shCXCL14*-expressing hESCs treated with or without CXCL14 (100 ng/ml). All error bars represent the standard deviations of three (H9) and five (S6) replicates. The significance level was set at *** $P < 0.001$; ANOVA (Tukey's multiple comparison test).

Figure S4

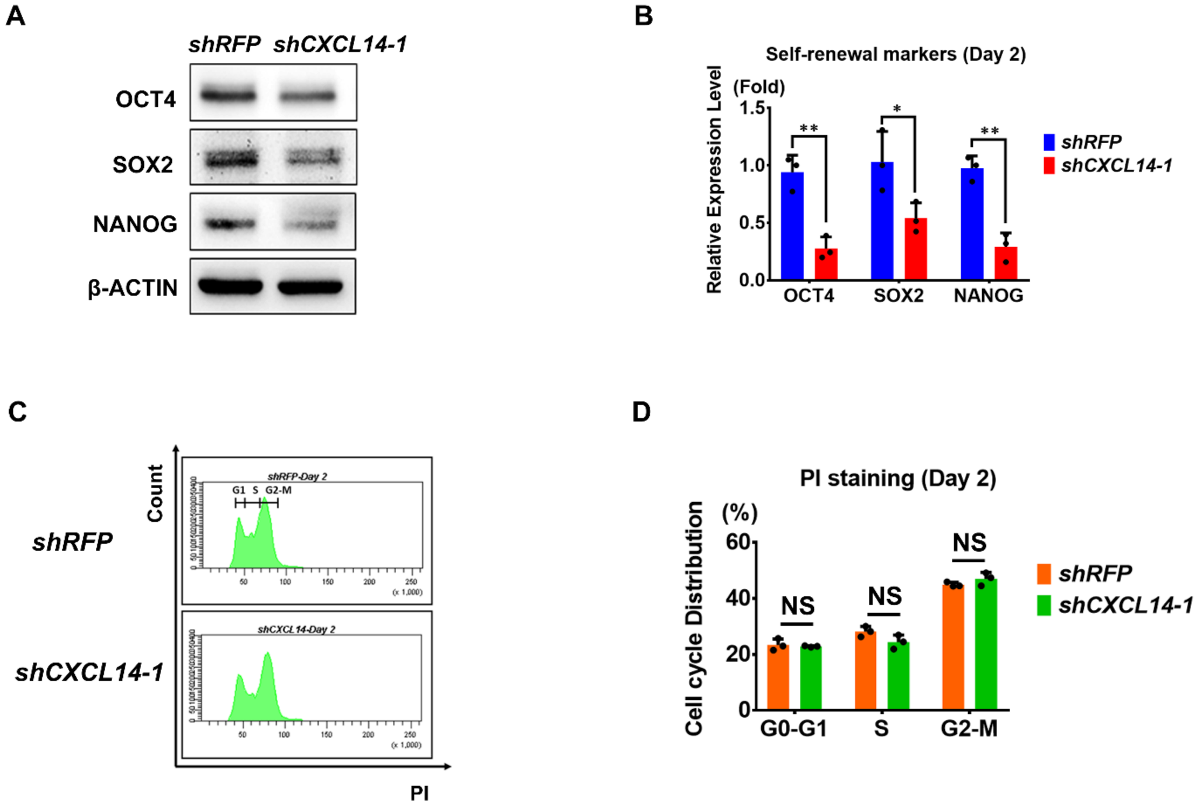

Figure S4. Downregulation of CXCL14 regulates hESC cell cycle through hESC differentiation. (**A**) Western analysis of self-renewal marker expression in knockdown of CXCL14 at the early time point (Day 2). β-ACTIN was applied as the internal control. (**B**) Quantification of western blot analysis A. The error bars represent the standard deviations of three replicates. The significance level was set at * $P < 0.05$, ** $P < 0.01$; Student's t-test. (**C**) PI staining with flow cytometry assay for cell cycle analysis of shRNA-infected cells at the early time point (Day 2). (**D**) Quantification data showing the distribution of G0-G1-phase, S-phase and G2-M-phase of *shRNA*-expressing hESCs at the early time point (Day 2). All error bars represent the standard deviations of three replicates. NS, no significant difference; Student's t-test.

Figure S5

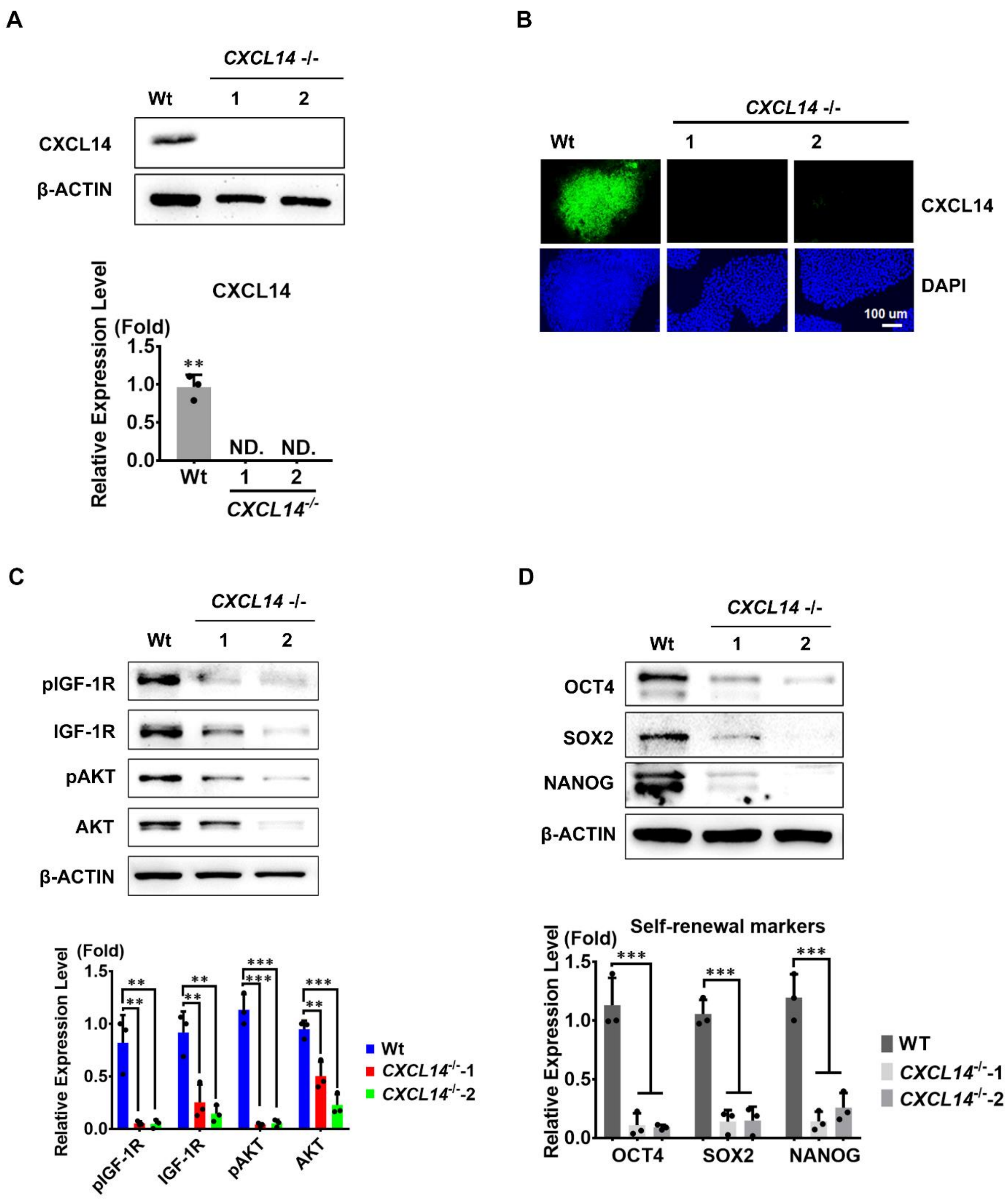

Figure S5. Knockout of *CXCL14* impairs self-renewal of hPSCs. (**A**) Western blot analysis and quantification of CXCL14 protein expression levels of WT and two independent *CXCL14*$^{-/-}$ hPSC lines. (**B**) Immunofluorescence staining for CXCL14 (green) protein expression levels of WT and *CXCL14*$^{-/-}$ hPSC lines. Cell nuclei were stained with DAPI (blue). The scale bar represents 100 μm. (**C**) Western blot analysis and quantification of phosphorylation of IGF-1R and the downstream activator AKT in WT and *CXCL14*$^{-/-}$ hPSC lines. (**D**) Western blot analysis and quantification of self-renewal markers OCT4, SOX2 and NANOG protein expression levels of WT and *CXCL14*$^{-/-}$ hPSC lines. β-ACTIN was

applied as an internal control. The error bars represent the standard deviations of three replicates. The significance level was set at ** $P < 0.01$, *** $P < 0.001$; ANOVA (Dunnett's multiple comparison test). ND, the data were non-detectable.

Table S1 qRT-PCR and sgRNA primer list.

Gene	Forward	Reverse
GAPDH	5' CATCACCATCTTCCAGGAGC 3'	5' ATGCCAGTGAGCTTCCCGTTC 3'
OCT4	5' AGCGAACCAGTATCGAGAAC 3'	5' TTACAGAACCACACTCGGAC 3'
SOX2	5' AGCTACAGCATGATGCAGGA 3'	5' GGTCATGGAGTTGTACTGCA 3'
NANOG	5' TGAACCTCAGCTACAAACAG 3'	5' TGGTGGTAGGAAGAGTAGAG 3'
CXCL14	5' AAGCTGGAAATGAAGCCAAA 3'	5' TGACCTCGGTACCTGGACAC 3'
IGF-1R	5' TTCAGCGCTGCTGATGTG 3'	5' AAGTTCCCGGCTCATGGT 3'
SOX1	5' GTACAGCATGATGATGGAGA 3'	5' CTGATCTCCGAGTTGTGCAT 3'
PAX6	5' ACATACCAAGCGTGTCATCAATAA 3'	5'ATGTTCTATTTCTTTGCAGCTTCC 3'
ZIC1	5' AAGCCCTTCAAGTGCGAGTTT 3'	5' GAACTGGGATGCGTGTAGGAC 3'
LHX5	5' GGCGAGGAGCTCTACGTCATC 3'	5' GACAAACTGCGGTCCGTACAG 3'
NOGGIN	5' TAGAGTTCTCCGAGGGCTTG 3'	5' CTCCGCAGCTTCTTGCTTAG 3'
BRYCHURY	5' TGCTTCCCTGAGACCCAGTT 3'	5' GATCACTTCTTTCCTTTGCATCAAG 3'
MIXL1	5′ CCGAGTCCAGGATCCAGGTA 3′	5′ CTCTGACGCCGAGACTTGG 3′
VIMENTIN	5' AAGCAGGAGTCCACTGAGTA 3'	5' AGTGTCTTGGTAGTTAGCAGC 3'
TWIST1	5' AGCTACGCCTTCTCGGTCT 3'	5' CCTTCTCTGGAAACAATGACATC 3'
SNAIL2	5' ACAGCGAACTGGACACACAT 3'	5' GATGGGGCTGTATGCTCCT 3'
SOX7	5′ GAACGCCTTCATGGTTTGGG 3′	5′ AGAGGGAGCTCAGAAGGAAGC 3′
SOX17	5' GGCGCAGCAGAATCCAGA 3'	5' CCACGACTTGCCCAGCAT 3'
AFP	5' AGCTTGGTGGTGGATGAA AC 3'	5' CCCTCTTCAGCAAAGCAGAC 3'
FOXA2	5' GGGAGCGGTGAAGATGGA 3'	5' TCATGTTGCTCACGGAGGAGTA 3'
GATA4	5' GGTCACTATCTGTGCAACGC 3'	5' GTTTGGATCCCCTCTTTCCG 3'
GATA6	5' GGATTGTCCTGTGCCAACTGTC 3'	5' TGACGCCTATGTAGAGCCCATC 3'
CXCL1	5' AGGGAATTCACCCCAAGAAC 3'	5' TGGATTTGTCACTGTTCAGCA 3'
CXCL2	5' GCAGGGAATTCACCTCAAGA 3'	5' GGATTTGCCATTTTTCAGCA 3'
CXCL3	5' GCAGGGAATTCACCTCAAGA 3'	5' GGTGCTCCCCTTGTTCAGTA 3'
CXCL4	5' AGCCTGGAGGTGATCAAGG 3'	5' CCATTCTTCAGCGTGGCTA 3'
CXCL5	5' GCAAGGAGTTCATCCCAAAA 3'	5' TTGTTTCCACCGTCCAAAAT 3'
CXCL6	5' GTCCTGTCTCTGCTGTGCTG3'	5' AACTTGCTTCCCGTTCTTCA 3'
CXCL7	5' TGTCATTGCTGCTGACTGCT 3'	5' CAAGTCACTGTCTAGACTTTCCTCTTT 3'
CXCL8	5' ACTGAGAGTGATTGAGAGTGGAC 3'	5' AACCCTCTGCACCCAGTTTTC 3'
CXCL9	5' TGTTCCCCTTTGCTTCATTC 3'	5' GAAAGGCACTGCATTGTGG 3'
CXCL10	5' GAAAGCAGTTAGCAAGGAAAGGT 3'	5' GACATATACTCCATGTAGGGAAGTGA 3'
CXCL11	5' AGTGTGAAGGGCATGGCTA 3'	5' TCTTTTGAACATGGGGAAGC 3'

CXCL12	5' ACCTCCTCTTTCAACCTCAGTG 3'	5' TCTCTGAGCACAGTCCCAGTAA 3'
CXCL13	5' CTCTGCTTCTCATGCTGCTG 3'	5' GCTCTCTTGGACACATCTACACC 3'
CXCL16	5' TGAGAGCTTACCATCGGTGTC 3'	5' TTGTTGCCTCCACACACG 3'
CXCL17	5' ACCGAGGCCAGGCTTCTA 3'	5' GGCTCTCAGGAACCAATCTTT 3'
**Gene**	**Forward**	**Reverse**
**sg*CXCL14-1***	5' TTGGGCGATAGCGGGTCCTTTCCCG 3'	5' AAACCGGGAAAGGACCCGCTATCGC 3'
**sg*CXCL14-2***	5' TTGGGGCGCTGTACACCGCGCGTG 3'	5' AAACCACGCGCGGTGTACAGCGCC 3'

Table S2 Primary antibody list.

Antibody	Company	Catalog	Used in
CXCL14	GeneTex	GTX52666	WB
CXCL14	Proteintech	10468-1-AP	IF
CXCL14	Cloud-Clone Corp.	PAB607Hu01	IP
TRA-1-60	Santa Cruz	sc-21705	IF
TRA-1-80	Santa Cruz	sc-21706	IF
SSEA-3	MybioSource	MBS555001	IF
SSEA-4	eBioscience	50-8843-82	IF
NANOG	Cell Signaling Technology	#4893	WB
NANOG	Santa Cruz	sc-33759	IF
SOX2	Cell Signaling Technology	#2748	WB
SOX2	GeneTex	N1C3	IF
OCT4	Santa Cruz	sc-9081	IF
OCT4	Cell Signaling Technology	#2890	WB
P21	Cell Signaling Technology	#2947	WB
P27	Cell Signaling Technology	#3686	WB
CDK1	Cell Signaling Technology	#9112	WB
CDK2	Cell Signaling Technology	#2546	WB
CDK6	Cell Signaling Technology	#3136	WB
IGF-1R	Cell Signaling Technology	#3027	WB/IP
IGF-1R	Proteintech	66283-1-AP	IF
pIGF-1R	Cell Signaling Technology	#3918	WB
His-probe	Santa Cruz	Sc-803	ELISA
β-ACTIN	Sigma-Aldrich	A5441	WB
AKT	Cell Signaling Technology	#9272	WB

pAKT	Cell Signaling Technology	#9271	WB
GATA4	Santa Cruz	sc-25310	IF
SOX17	Millipore	09-038-I	IF
SOX7	Proteintech	23925-1-AP	IF
Ki-67	Boster	M00254-4	IF
pHISTONE H3	Millipore	06-570	IF

WB= Western Blot IF= Immunoflurescence IP= Immunoprecipitation.
